# Clinical characteristics and chest computed tomography findings related to the infectivity of pulmonary tuberculosis

**DOI:** 10.1186/s12879-021-06901-2

**Published:** 2021-11-27

**Authors:** Yuanyuan Wang, Xiaoqian Shang, Liang Wang, Jiahui Fan, Fengming Tian, Xuanzheng Wang, Weina Kong, Jing Wang, Yunling Wang, Xiumin Ma

**Affiliations:** 1 First Affiliated Hospital of Xinjiang Medical University, Urumqi, 830011 Xinjiang People’s Republic of China; 2 State Key Laboratory of Pathogenesis, Prevention and Treatment of High Incidence Diseases in Central Asia, Clinical Laboratory Center, Tumor Hospital Affiliated to Xinjiang Medical University, Urumqi, 830011 Xinjiang People’s Republic of China; 3 Respiratory Department of the Second Affiliated Hospital of Hainan Medical College, Haikou, 570000 Hainan People’s Republic of China

**Keywords:** Pulmonary tuberculosis, Acid-fast bacilli-negative, Acid-fast bacilli-positive

## Abstract

**Aim:**

This study mainly evaluates the clinical characteristics and chest chest computed tomography (CT) findings of AFB-positive and AFB-negative pulmonary tuberculosis (PTB) patients to explore the relationship between AFB-positive and clinico-radiological findings.

**Methods:**

A retrospective analysis of 224 hospitalized tuberculosis patients from 2018 to 2020 was undertaken. According to the AFB smear results, they were divided into AFB-positive pulmonary tuberculosis (positive by Ziehl–Neelsen staining) and AFB-negative pulmonary tuberculosis and patients’ CT results and laboratory test results were analyzed.

**Results:**

A total of 224 PTB patients were enrolled. AFB-positive (n = 94, 42%) and AFB-negative (n = 130, 58%). AFB-positive patients had more consolidation (77.7% vs. 53.8%, p < 0.01), cavity (55.3% vs. 34.6%, p < 0.01), calcification (38.3% vs. 20%, p < 0.01), bronchiectasis (7.5% vs. 1.5%, p < 0.05), bronchiarctia (6.4% vs. 0.8%, p < 0.05), and right upper lobe involvement (57.5% vs. 33.1%, p < 0.01), left upper lobe involvement (46.8% vs. 33.1%, p < 0.05) and lymphadenopathy (58.5% vs. 37.7%, p < 0.01).

**Conclusion:**

The study found that when pulmonary tuberculosis patients have consolidation, cavity, upper lobe involvement and lymphadenopathy on chest CT images, they may have a higher risk of AFB-positive tuberculosis.

## Introduction

Mycobacterium tuberculosis (Mtb) is a highly survivable intracellular bacterium that causes tuberculosis (TB), which infects about one-third of the world's population and poses a major threat to human health [1]. Because of its large population base, China has a high incidence of TB, second only to India in terms of the total number of cases [2]. Studies have shown [3] that the prevalence of pulmonary tuberculosis (PTB) in China differs greatly between eastern and western regions, with the prevalence in western regions significantly higher than in other regions. Most patients are concentrated in remote areas of China such as Xinjiang. PTB is a widespread disease that seriously threatens the lives and health of residents in Xinjiang and restricts social and economic development. It is considered a social and public health problem [4–7].

The most susceptible site of Mtb infection is the lung [8]. Prompt diagnosis of PTB is essential for effective treatment and infection control. In order to diagnose PTB, microscopic examination and culture of respiratory tract specimens (sputum or bronchial fluid) are recommended [9]. Although Mtb culture is most sensitive to find acid-fast bacilli (AFB), it’s time period is 2 to 12 weeks, thus for the initial diagnosis of PTB, the effectiveness is considered to be limited. Owing to the short processing time of Ziehl–Neelsen (ZN) staining, it can be used for initial diagnosis of PTB. At the same time, ZN staining of great importance for assessing the degree of infectivity. AFB-positive tuberculosis patients have higher infectivity than AFB-negative patients, so the infectivity of PTB is directly related to the positive results of the smear. Therefore, patients with AFB-positive PTB have to be placed in a separate isolation ward to prevent the spread of the disease [10].

Imaging is one of the most critical diagnostic assessments of PTB [11]. Compared with chest X-ray [12], computed tomography (CT) is more sensitive in the detection of microscopic solid tuberculosis processes. However, few studies have reported the CT findings of AFB-positive compared to AFB-negative PTB. Considering the huge social pressure brought by tuberculosis, this study aims to evaluate the clinical characteristics and CT manifestations of AFB-positive and AFB-negative PTB in hospitalized adult patients.

## Methods

### Patients

First, this was a retrospective study. The First Affiliated Hospital of Xinjiang Medical University is a tertiary first-class hospital. The study reviewed adult patients (≥ 18 years of age) who were diagnosed with PTB in the hospital's inpatient ward from 2018 to 2020. According to the AFB smear results, they are divided into AFB-positive group (Ziehl–Neelsen staining is positive) and AFB-negative group. Chest CT scan is required for all patients (only include patients within 14 days of diagnosis of PTB). Patient demographic data, primary medical status, AFB smear microscopic results, CT findings, and laboratory results were collected and analyzed.

PTB patients included in the study were those that conform to the PTB diagnosis standard "WS288-2017 Tuberculosis Diagnosis" [13] formulated by China in 2018. They have symptoms of PTB, and meet at least one of the following items: (1) Sputum Mtb test is positive (including smear or culture); (2) The sputum Mtb was negative, but the chest imaging examination showed typical manifestations of active TB; (3) The pathological diagnosis of pulmonary lesion specimens or pleural fluid and bronchoalveolar lavage fluid was tuberculosis. (4) Exclude patients with other immune and tumor diseases.

### Details of CT

All chest CT were performed by a 128-slice dual-source CT (Somatom Definition Flash, Siemens Healthcare, Forchheim, Germany) with intravenous contrast. When the patient is in the supine position, the image is acquired during inhalation. Transverse and coronal CT images of the chest were reconstructed using Syngo software. The reconstructed chest CT image was analyzed on PACS (Picture Archiving and Communication Systems). The results of the chest CT were examined by two CT physicians and various features of the PTB were scored. The final result was performed by a radiologist with 15 years of experience in interpretation of chest CT results.

### Statistical analysis

Clinical data, CT findings and laboratory results were compared between the AFB-negative and AFB-positive PTB cases. All experimental data in this study were statistically analyzed using SPSS 22.0 (Inc., Chicago, IL) and GraphPad Prism 8 software. Quantitative data conforming to normal distribution and homogeneous variance were expressed as mean ± standard deviation ($\overline{x} \pm s$). Pearson chi-square test or Fisher's exact test were used for dichotomous variables. p < 0.05 was considered statistically significant.

## Results

### Demographic information of PTB

A total of 224 PTB patients were enrolled in the study. Table 1 list detailed demographic information. Among them, 111 were males and 113 were females, with an average age of 55.60 ± 15.86 years (18 to 81 years), and more patients were over 45 years old.

**Table 1:** Demographic characteristics of hospitalized adult patients with PTB

	PTB n = 224 (%)	AFB negative n = 130 (%)	AFB positive n = 94 (%)	P
Sex				0.23
Male	111 (49.5)	60 (46.2)	51 (54.3)	
Female	113 (50.5)	70 (53.8)	43 (45.7)	
Age (years)	55.60 ± 15.86	50.41 ± 15.95	51.13 ± 16.13	0.74
16–18	8 (3.6)	3 (2.3)	4 (4.2)	
19–30	19 (8.5)	11 (8.5)	8 (8.6)	
31–45	51 (22.8)	31 (23.8)	20 (21.2)	
≥ 45	146 (65.1)	85 (65.4)	63 (67)	
Ethnicity				0.21
Han	144 (64.3)	88 (67.7)	56 (59.6)	
Minority	80 (35.7)	42 (32.3)	38 (40.4)	
Degree of education				0.89
Primary	43 (19.2)	27 (20.8)	16 (17)	
Middle	97 (43.3)	55 (42.3)	42 (44.7)	
College	60 (26.8)	35 (26.9)	25(26.6)	
Smoking history	63 (28.1)	26 (20)	37 (39.4)	< 0.01*
Drinking history	40 (17.9)	17 (13)	23 (3.2)	0.03^*^
HIV Positive	1 (0.5)	1 (0.8)	0	0.39
BCG scars	202 (90.2)	119 (91.5)	83 (88.3)	0.42
Previous PTB	28 (12.5)	17 (13.1)	11 (11.7)	0.76
Chronic disease				
Heart disease	24 (10.7)	14 (10.8)	10 (10.6)	0.97
Pulmonary disease	18 (8)	13 (10)	5 (5.3)	0.20
Liver disease	6 (2.7)	4 (3.1)	2 (2.1)	0.66
Kidney disease	3 (1.3)	1(0.8)	2 (2.1)	0.38
Neurologic disease	1 (0.4)	1 (0.8)	0	0.39
Diabetes	36 (16.1)	15 (11.5)	21 (22.3)	0.03^*^
Hypertension	51 (22.8)	27 (20.8)	24 (25.5)	0.23

*PTB* pulmonary tuberculosis, *AFB* acid-fast bacilli, *HIV* human immunodeficiency virus, *BCG* Bacillus Calmette–Guerin; Note: * < 0.05

The PTB patients were categorized in two groups of AFB-positive and AFB-negative. AFB-positive PTB patients with a history of smoking (p < 0.01) and drinking (p = 0.03) are more than among AFB-negative patients. More than one-fifth of AFB-positive PTB patients had diabetes, more than AFB-negative patients (P = 0.03). However, the two groups were not significantly different with respect to age, gender distribution, ethnicity or degree of education.

### Clinical characteristics of PTB

Table 2 lists the clinical characteristics of PTB patients. In this study, AFB-positive patients had more symptoms of cough, fever, hyperhidrosis and fatigues than AFB-negative patients (p < 0.01). However, there were no significant differences between hemoptysis and nutrition between the two groups.

**Table 2:** Clinical symptoms of hospitalized adult patients with PTB

	PTB n = 224 (%)	AFB negative n = 130 (%)	AFB positive n = 94 (%)	p
Cough	122 (54.5)	60 (46.1)	62 (66)	< 0.01^*^
Fever	56 (25)	13 (10)	43 (45.7)	< 0.01^*^
Hyperhidrosis	31 (13.8)	7 (5.4)	24 (25.5)	< 0.01^*^
Fatigue	49 (21.9)	17 (13.1)	32 (34)	< 0.01^*^
Hemoptysis	10 (4.5)	4 (3.1)	6 (6.4)	0.31
Nutrition				
Normal weight	130 (58)	76 (58.5)	54 (57.5)	0.88
Underweight (BMI < 18.5 kg/m^2^)	14 (6.3)	9 (6.9)	5 (5.3)	0.62
Overweight (BMI > 25 kg/m^2^)	80 (35.7)	45 (34.6)	35 (37.2)	0.69

*PTB* pulmonary tuberculosis, *AFB* acid-fast bacilli; Note: * < 0.05.

### CT findings

**Fig. 1:**
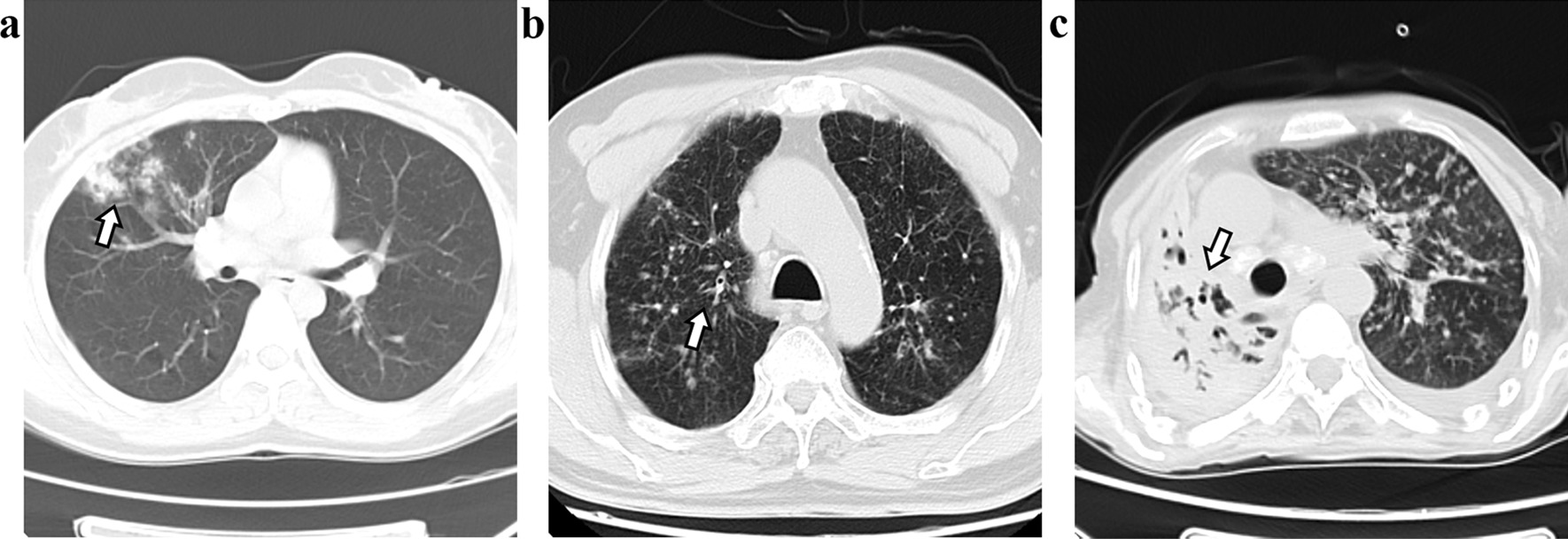
Representative CT findings of hospitalized adult patients with PTB. **a** AFB-negative, CT findings show multiple nodules in the upper lobes of both lungs, and the nodules distributed along the lungs show a "tree bud sign" (arrow); **b** AFB-negative, multiple patches and nodules in the right middle lobe (arrow); **c** AFB-positive, large consolidation of the right lung, worm-eaten cavities (arrows) visible inside, scattered nodules in the left lung, and mediastinum shifted to the right

Among 224 cases of PTB, the most common CT findings were nodular lesions (96%), followed by consolidation (63.8%) and cavity lesions (43.3%). In most patients, the right upper lobe (43.3%) and the left lower lobe were affected (42.9%). Compared with AFB-negative, AFB-positive had more consolidation (53.8% vs 77.7%, p < 0.01), cavity (34.6% vs 55.3%, p < 0.01), calcification (20% vs 38.3%, p < 0.01), bronchiectasis (1.5% vs 7.5%, p = 0.02), bronchiarctia (0.8% vs 6.4%, p = 0.01), right upper lobe involvement (33.1% vs 57.5%, p < 0.01), and left upper lobe involvement (33.1% vs 46.8%, p = 0.03). In addition, in AFB-positive, lymphadenopathy was more common (37.7% vs 58.5%, P < 0.01) (Table 3). AFB-negative CT findings showed multiple nodules in both lungs, and the nodules were "tree buds" or "patches" (Fig. 1a, b). However, AFB-positive CT findings showed more consolidation and cavities (Fig. 1c). In a multivariate logistic regression analysis using variables with a p < 0.2 on comparison analysis, CT findings that AFB-positive PTB is more likely to have consolidation than AFB-negative PTB was the only statistically significant difference between groups.

**Table 3:** CT results of hospitalized adult patients with PTB

	AFB negative n = 130 (%)	AFB positive n = 94 (%)	P
Parenchymal lesion			
Consolidation	70 (53.8%)	73 (77.7%)	< 0.01^*^
Cavity	45 (34.6)	52 (55.3)	< 0.01^*^
Nodule	125 (96.2)	90 (95.7)	0.88
Ground-glass opacity	13 (10)	16 (17)	0.12
Bronchiectasis	2 (1.5)	7 (7.5)	0.02^*^
Bronchiarctia	1 (0.8)	6 (6.4)	0.01^*^
Calcification	26 (20)	36 (38.3)	< 0.01^*^
Lesion location			
Right upper lobe (case)	43 (33.1)	54 (57.5)	< 0.01^*^
Right middle lobe (case)	39 (30)	36 (38.3)	0.19
Right lower lobe (case)	50 (38.5)	41 (43.6)	0.44
Left upper lobe (case)	43 (33.1)	44 (46.8)	0.03^*^
Left lower lobe (case)	54 (41.5)	42 (44.7)	0.64
Non-parenchymal lesion			
Pleural effusion	26 (20)	23 (24.5)	0.42
Pleural thickening	38 (29.2)	34 (36.2)	0.27
Lymphadenectasis	49 (37.7)	55 (58.5)	< 0.01^*^

*AFB* acid-fast bacilli; Note: * < 0.05

### Laboratory results of PTB

Table 4 outlines the laboratory results. Compared with AFB-negative patients, AFB-positive patients more often had low-level aspartate aminotransferase (AST) and low-level glutamate pyruvic transaminase (ALT) (7.7% vs 17%, p = 0.03; 1.5% vs 7.5%, p = 0.03), leukocytosis (12.3% vs 22.3%, p = 0.04), reduced lymphocytes (29.8% vs 18.5%, p = 0.04), increased IL-6 (24.6% vs 37.2%, p = 0.04), and increased CRP (26.9% vs 39.4%, p = 0.04). The above data show that lower AST, ALT and lymphocytes, higher leukocytosis, CRP and IL-6 may be important laboratory indicators.

**Table 4:** Laboratory results of hospitalized adult patients with PTB

	AFB negative n = 130 (%)	AFB positive n = 94 (%)	p
Albumin (< 40 g/l)	57 (43.9)	50 (53.2)	0.17
Anemia (Hb: male < 140 g/l, Female < 120 g/l)	52 (40)	39 (41.5)	0.82
AST			
Increased (> 35 u/l)	10 (7.7)	7 (7.5)	0.94
Decreased (< 13 u/l)	10 (7.7)	16 (17)	0.03^*^
ALT			
Increased (> 40 u/l)	10 (7.7)	9 (9.6)	0.62
Decreased (< 7 u/l)	2 (1.5)	7 (7.5)	0.03^*^
Leukocytosis (white blood cells > 9.5 × 109/l)	16 (12.3)	21 (22.3)	0.04^*^
Lymphocytosis (lymphocytes > 3.2 × 109/l)	3 (2.3)	5 (5.3)	0.23
Lymphopenia (lymphocytes < 1.1 × 109/l)	24 (18.5)	28 (29.8)	0.04^*^
Monocyte (monocytes > 0.6 × 109/l)	36 (27.7)	27 (28.7)	0.86
RBC (red blood cells < 4.3 × 1012/l)	41 (31.5)	26 (27.7)	0.53
Hct (< 40%)	59 (45.4)	36 (38.3)	0.29
Thrombocytosis (platelets > 350 × 109 /l)	12 (9.2)	13 (13.8)	0.28
PCT (> 0.05 ng/ml)	26 (20)	23 (24.5)	0.42
IL-6 (> 7 pg/ml)	32 (24.6)	35 (37.2)	0.04^*^
CRP (> 8 mg/l)	35 (26.9)	37 (39.4)	0.04^*^
T-SPOT.TB Positive (> 0.35 IU/ml)	41 (31.5)	33 (35.1)	0.57

*AFB* acid-fast bacilli, *AST* aspartate aminotransferase, *ALT* glutamate pyruvic transaminase, *RBC* white blood cells, *Hct* red blood cell specific volume, *PCT* procalcitonin, *IL-6* interleukin-6, *CRP* C-reactive protein; Note: * < 0.05

## Discussion

In this study, Han nationality was more likely to have PTB than ethnic minority groups, and this difference was mostly observed in patients > 45 years old. In our country's immunization program, since 1949, all people should be vaccinated with BCG. Therefore, 202 of the subjects in this study had been vaccinated with BCG. This measure is considered to greatly reduce the probability of tuberculosis infection [14]. But it also suggested that BCG vaccination cannot completely prevent tuberculosis infection. Among 224 cases of PTB, about 1/5 of the patients smoked and drank alcohol. The number of AFB-positive PTB patients with a history of smoking was significantly higher than among AFB-negative PTB patients. This interesting finding suggested that not only smoking and drinking were common in PTB patients, but there were also differences in different groups. The study found that AFB-positive PTB also have more diabetes, which was consistent with the results of previous studies that PTB patients are more likely to develop diabetes [15].

When PTB occurs, there are often obvious clinical symptoms [16], such as cough, fever, hyperhidrosis, fatigue, hemoptysis, etc. Compared with AFB-negative PTB, the study found that cough, fever, hyperhidrosis and fatigue are more frequent in AFB-positive patients. Badawi et al. [17] found that PTB patients lost significant weight. However, PTB patients in this study did not lose significant weight and instead gained more weight (Table 2). It may be because Xinjiang (China) is a livestock area, and people eat more dairy and meat products, which has a significant impact on the weight of PTB patients.

In AFB-positive PTB, CT findings such as consolidation, cavity, nodular lesions, upper lobe involvement and lymphadenectasis were more frequent. These results suggest that the results of specific CT tests are related to the infectivity of PTB. CT findings of consolidation, cavity, and multilobe involvement were important findings of AFB-positive PTB, which are consistent with previous studies [18]. Caseous necrosis is the main findings of consolidation. The degree of consolidation was closely related to the amount of AFB, since AFB is abundant in caseous necrotic tissue. In addition, Khan [19] that the number of consolidated lung lobes increased with the number of AFB, suggesting that PTB had intrabronchial spread. Therefore, the results of this study once again confirmed the importance of consolidation and multilobe involvement in AFB-positive PTB.

Lymphadenectasis is another important finding of AFB-positive PTB. Mediastinal lymph nodes are the necessary sites for tuberculosis infection to spread from the pulmonary parenchyma [20], and are important sites for the persistence of a large number of AFB. Therefore, lymphadenectasis may be one of the indicators of PTB development. In this study, there was a significant increase in upper lobe involvement in AFB-positive PTB. Nodular lesions are considered to be the most common CT finding of PTB. However, the presence of nodular lesions was not associated with AFB-positive PTB. It may be related to the small number of AFB in nodular lesions and the longer distance from the central airway, therefore, patients with nodular lesions are not highly infectious.

Of course, our research has some limitations. First, this was a single-center retrospective study. Therefore, the selection bias of a small number of heterogeneous patients may have affected our analysis. Second, unmeasured variables may have other effects. The results of this study did not show the importance of underlying disease in a multivariate analysis of the association with AFB-positive PTB.

## Conclusion

AFB-positive PTB is highly infectious, which seriously affects the quality of life and health of residents in Xinjiang. The study found that AFB-positive PTB patients had more frequent performances symptoms of cough, fever, hyperhidrosis and fatigue. At the same time, when PTB patients have consolidation, cavity, upper lobe involvement and lymphadenopathy on chest CT images, they may have a higher risk of AFB-positive tuberculosis. In order to assess the infectivity of PTB in time, it is necessary to conduct prospective studies on more patients.

## Data Availability

The data used to support the findings of this study are available from the corresponding author upon request.

## Abbreviations

PTB: Pulmonary tuberculosis
Mtb: Mycobacterium tuberculosis
CT: Computed tomography
AFB: Acid-fast bacilli
HIV: Human immunodeficiency virus
BCG: Bacillus Calmette–Guerin
AST: Aspartate aminotransferase
ALT: Glutamate pyruvic transaminase
RBC: White blood cells
Hct: Red blood cell specific volume
PCT: Procalcitonin
IL-6: Interleukin-6
CRP: C-reactive protein
